# Safety and tolerability of pirfenidone in asbestosis: a prospective multicenter study

**DOI:** 10.1186/s12931-022-02061-2

**Published:** 2022-05-28

**Authors:** Jelle R. Miedema, Catharina C. Moor, Marcel Veltkamp, Sara Baart, Natascha S. L. Lie, Jan C. Grutters, Marlies S. Wijsenbeek, Rémy L. M. Mostard

**Affiliations:** 1 Department of Pulmonology, Center of Excellence for Interstitial Lung Disease and Sarcoidosis, Erasmus University Medical Center, Rotterdam, The Netherlands; 2 Department of Pulmonology, ILD Center of Excellence, St. Antonius Hospital, Nieuwegein, The Netherlands; 3 Division of Heart and Lungs, University Medical Center, Utrecht, The Netherlands; 4 Department of Biostatistics, Erasmus MC, Rotterdam, The Netherlands; 5 Department of Pulmonology, Zuyderland Medical Center, Heerlen, Sittard, The Netherlands

**Keywords:** Asbestosis, Pirfenidone, Anti-fibrotic treatment, Safety, Efficacy, Home monitoring

## Abstract

**Background:**

Pirfenidone slows down disease progression in idiopathic pulmonary fibrosis (IPF). Recent studies suggest a treatment effect in progressive pulmonary fibrosis other than IPF. However, the safety and effectiveness of pirfenidone in asbestosis patients remain unclear. In this study, we aimed to investigate the safety, tolerability and efficacy of pirfenidone in asbestosis patients with a progressive phenotype.

**Methods:**

This was a multicenter prospective study in asbestosis patients with progressive lung function decline. After a 12-week observational period, patients were treated with pirfenidone 801 mg three times a day. Symptoms and adverse events were evaluated weekly and patients completed online patient-reported outcomes measures. At baseline, start of therapy, 12 and 24 weeks, in hospital measurement of lung function and a 6 min walking test were performed. Additionally, patients performed daily home spirometry measurements.

**Results:**

In total, 10 patients were included of whom 6 patients (66.7%) experienced any adverse events during the study period. Most frequently reported adverse events were fatigue, rash, anorexia and cough, which mostly occurred intermittently and were reported as not very bothersome. No significant changes in hospital pulmonary function (forced vital capacity (FVC), diffusion capacity of the lung for carbon monoxide (DLCO), 6 min walking test or patient-reported outcomes measures before and after start of pirfenidone were found. Home spirometry demonstrated a FVC decline in 12 weeks before start of pirfenidone, while FVC did not decline during the 24 week treatment phase, but this difference was not statistically significant.

**Conclusions:**

Treatment with pirfenidone in asbestosis has an acceptable safety and tolerability profile and home spirometry data suggest this antifibrotic treatment might attenuate FVC decline in progressive asbestosis.

*Trial registration* MEC-2018-1392; EudraCT number: 2018-001781-41

**Supplementary Information:**

The online version contains supplementary material available at 10.1186/s12931-022-02061-2.

## Background

Asbestosis is a rare occupational interstitial lung disease, caused by inhalation of asbestos fibers [1]. Although the use of asbestos has been restricted or banned in many countries, global incidence of asbestosis has increased, including in Western Europe and North America [2]. The disease course is variable, but a significant subgroup of asbestosis patients have a progressive fibrotic phenotype [3, 4]. Recently, a retrospective study in Germany described the course of lung function in asbestosis patients and found progressive disease, defined as an annual forced vital capacity (FVC) loss of 100 ml or more in 20% of subjects [3]. Another study assessed the gender, age and physiologic (GAP) variables model to predict survival and found a high 3-year mortality risk of 33.3% and 60.0% for GAP stage II and III asbestosis respectively [4]. The use of immunosuppressive drugs is not recommended and there is no specific treatment available for these patients [1]. Therefore, new therapeutic strategies in progressive asbestosis are highly warranted.

Pirfenidone is an antifibrotic drug that has been used extensively in idiopathic pulmonary fibrosis (IPF). It slows down lung function decline and post-hoc analyses also show a positive effect on mortality and risk of respiratory related hospitalizations [5, 6]. Asbestosis shows many similarities with idiopathic pulmonary fibrosis (IPF), including demographic characteristics of patients and genetic risk factors [7, 8]. Overlapping pathways and mechanisms in IPF and other diseases that manifest with progressive pulmonary fibrosis have been hypothesized, independent of the underlying disease [9]. It has been demonstrated that nintedanib is effective in slowing down disease progression in patients with progressive pulmonary fibrosis other than IPF [10]. Additionally, two phase 2 studies on the treatment of pulmonary fibrosis suggested that pirfenidone slows down decline of FVC in unclassifiable pulmonary fibrosis and progressive fibrotic ILD other than IPF [11, 12]. Therefore, pirfenidone could be a promising treatment strategy in progressive asbestosis. Antifibrotic therapy has not been specifically evaluated in asbestosis patients and only three asbestosis patients were treated with pirfenidone in one of these studies [11].

In this small exploratory study, we aimed to prospectively investigate the safety and tolerability of pirfenidone in asbestosis patients with a progressive phenotype. Secondary objectives were to evaluate the efficacy of pirfenidone in asbestosis measured by home and hospital spirometry, and to assess changes in (health-related) quality of life during treatment.

## Methods

### Study design and participants

This was a multicenter prospective study at three sites in the Netherlands performed by the Dutch Association of Pulmonologists (NVALT). Ethics approval was obtained in all participating sites (MEC-2018-1392). All patients provided written informed consent before start of the study. To be eligible for inclusion, a combination of (1) previous asbestos exposure with a proper latency period, (2) pulmonary fibrosis and (3) pleural plaques or confirmation of asbestos fibers in lung biopsy or bronchoalveolar lavage was defined, in line with the American Thoracic Society statement on diagnosis of nonmalignant asbestos related disease [1]. The diagnosis of asbestosis was subsequently confirmed by central review expert panel of the NVALT. Further inclusion criteria were: age between 40 and 85 years, FVC ≥ 50% of predicted, diffusion capacity of the lung for carbon monoxide (DLCO) ≥ 25% of predicted, a FEV1/FVC ratio of > 0.7, a minimal 6 min walking distance of 150 m, > 10% interstitial fibrosis on HRCT by visual scoring of an experienced thoracic radiologist, and finally, documented disease progression within 6 months prior to the study. Disease progression was defined as FVC decline > 5% or DLCO decline of > 10% or decrease of > 25 m on 6 min walking test during the last 6 months. Patients who were treated with immunosupressants except prednisone ≤ 10 mg per day were excluded. Other exclusion criteria can be found in Additional file 1.

### Study procedures

After inclusion in the study, a 12-week observational period without the study drug was started. During this period patients were asked to perform daily home spirometry (FVC) measurements. At the baseline study visit, patients received a Bluetooth-enabled handheld spirometer (Spirobank Smart, MIR, Italy), connected with the CE-marked online application “ILD online” (Gezondheidsmeter, Curavista, the Netherlands). The application was pre-installed on a password-protected tablet computer. All results were directly transmitted to the hospital via a secure encrypted connection, which enables patients and investigators to access and review data directly. Next to home spirometry measurements, patients were asked to report weekly symptoms and adverse events in the online application.

After 12 weeks of observation, all included patients were started on pirfenidone (Esbriet capsules 267 mg or 801 mg), starting with 267 mg three times a day for 1 week, 534 mg three times a day for 1 week, followed by 801 mg three times a day as a maintenance dose for a total treatment period of 24 weeks. Study visits were planned at baseline, start of treatment, 12, and 24 weeks after start of treatment with pirfenidone. At study visits, patients performed lung function measurements (FVC and DLCO) and a 6 min walking test (6MWD). During the treatment period patients continued the daily home spirometry measurements. Routine laboratory test were performed monthly during the treatment period. During the treatment phase, patients were actively asked whether they experienced any (pre-specified) side-effects via the online application every week. In addition, patients could also report other side-effects.

At baseline, start of therapy, and 12 and 24 weeks after start of study treatment, patients completed two validated patient-reported outcomes measures online. The King’s Brief Interstitial Lung Disease (K-BILD) is a 15-item validated health status questionnaire with three domains (psychological, breathlessness and activities, and chest symptoms). Scores range from 0 to 100 with higher scores representing a better health status. The Leicester Cough Questionnaire (LCQ) is a 19-item questionnaire on cough-related quality of life. Total scores range from 3 to 21 with higher scores indicating a better cough-related quality of life.

### Statistical analysis

The primary outcome of safety and tolerability was recorded descriptively (number and percentage). Home spirometry data were analyzed with a piecewise linear mixed model, to examine differences in lung function decline between the observation and treatment period. In-hospital lung function measurements were analyzed descriptively (median, interquartile range). As this was a descriptive safety study, a formal power calculation was not possible. Based on feasibility, we aimed to include 10 patients.

## Results

Between April 2019 and June 2020, 10 patients were included. Two patients died during the study. One patient died during the observation period due to progression of his asbestosis, the other patients died as a result of euthanasia during the treatment period of the study. All patients were male, ex-smoker, mean age was 74.7 years (SD 7.2). Median FVC at baseline was 2.97L (IQR 2.85–3.69) or 73.0% of predicted (IQR 68.8–96.0), and median DLCO was 42.5% of predicted (IQR 40–49) (*n* = 8). One patient used supplemental oxygen at baseline. Most common comorbidity was cardiovascular disease in 50% of patients.

### Safety and tolerability

In total, 6 patients (66.7%) experienced any intermittent adverse event during the study period. Most frequently reported adverse events were fatigue, rash, anorexia and cough (Table 1). All patients were able to continue treatment during the study. However, in four patients pirfenidone dosage was reduced due to gastrointestinal side effects (n = 2), skin rash (n = 1) and dizziness (n = 1) with good clinical effect, enabling patients to continue the treatment. In two patients, pirfenidone was temporarily reduced due to skin rash, which resolved after treatment and the patients were able to continue with 801 mg three times a day. No dose adjustments had to be made due to elevation of aminotransferases, bilirubin or other abnormalities in laboratory tests. Two patients were hospitalized during the study, one due to pneumonia, and one to angina, not related to the treatment.

**Table 1:** Weekly self-reported adverse events

Adverse events	Number of patients (%)
Fatigue	5 (55.6)
Rash	4 (44.4)
Anorexia	4 (44.4)
Cough	4 (44.4)
Headache	3 (33.3)
Insomnia	3 (33.3)
Dizziness	3 (33.3)
Nausea	3 (33.3)
Dyspepsia	3 (33.3)
Decrease in weight	2 (22.2)
Flatulence	1 (11.1)

### In-hospital pulmonary function

**Fig. 1:**
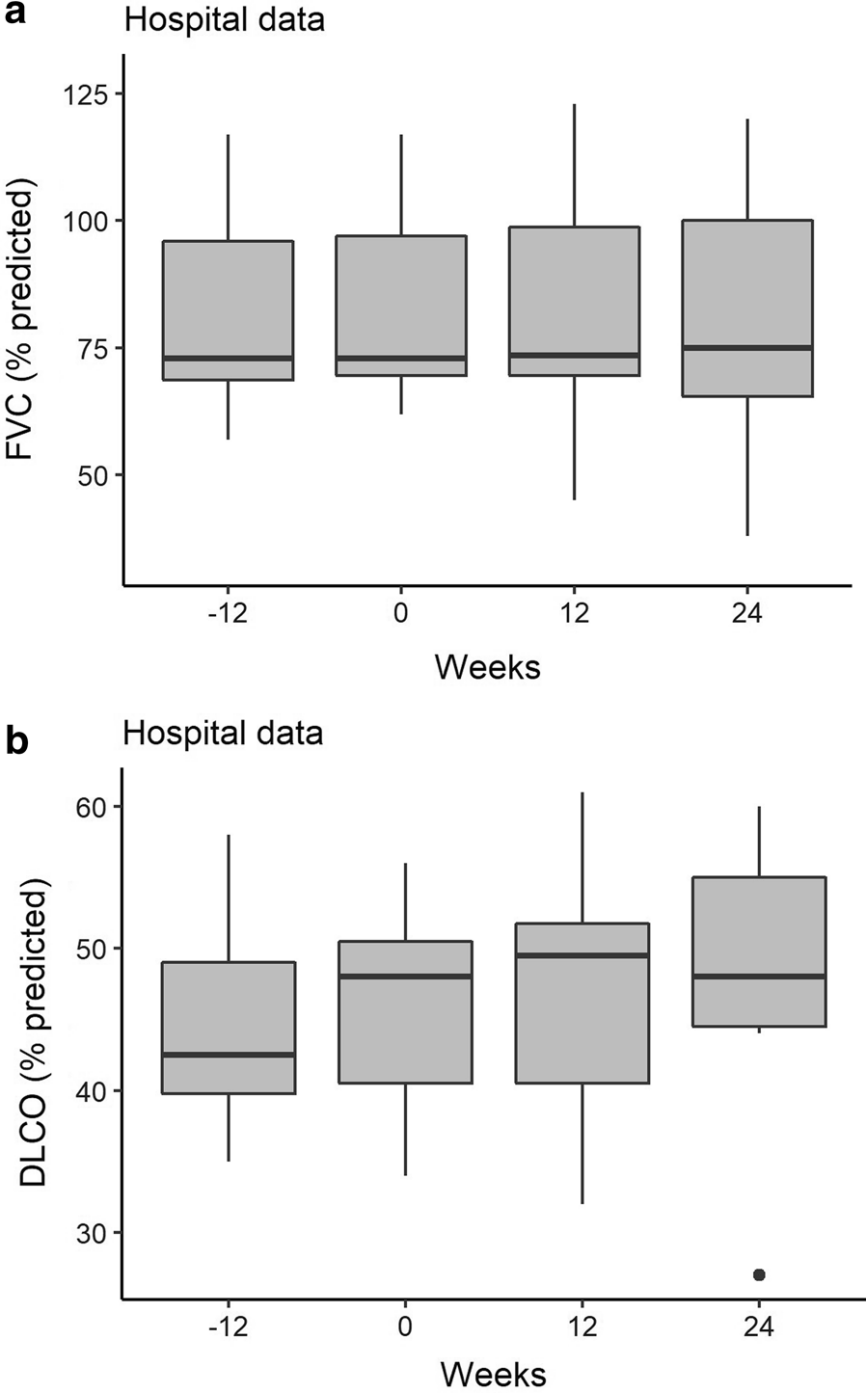
**a** Boxplots of forced vital capacity (FVC) % predicted and **b** diffusion capacity of the lungs for carbon monoxide (DLCO) % predicted, measured in hospital at start of the study, start of treatment with pirfenidone, 12 and 24 weeks after start of treatment. In total, n = 8 patients were included in the analysis of in-hospital measurements. At t = 0 and t = 24 weeks, pulmonary function data from n = 7 patients were available for analysis

In-hospital pulmonary function (FVC and DLCO), measured at baseline, start of pirfenidone, 12 and 24 weeks after start of treatment in 8 patients, remained stable before and during treatment (Fig. 1). Baseline 6 min walking test showed a median distance of 405 m (IQR 335–448) and did not change significantly during the study period.

### Home spirometry

**Fig. 2:**
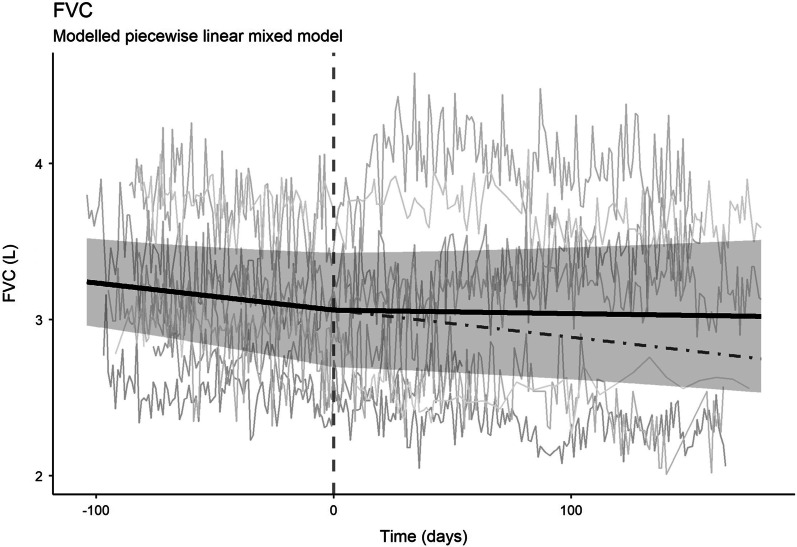
Repeated home spirometry FVC measurements in n = 9 individual patients (grey lines) and measured median FVC slope (black line) with 95% CI (grey area), before and after initiation of pirfenidone (time = 0). The dotted black line demonstrates the estimated continued FVC slope line without treatment

In the observation period, FVC significantly declined (slope − 0.0017, SE 0.009, p = 0.047). After start of treatment, FVC remained stable (slope − 0.00021, SE 0.0007, p = 0.76). The slopes before and after start of treatment did not significantly differ (p = 0.14). Slopes of FVC (in liters) over time and corresponding 95% CI are displayed in Fig. 2. Data from the patient who died before start of treatment were excluded from this analysis.

### Patient reported outcome measures (PROMs)

At baseline, mean K-BILD score was 46.8 (SD 7.5) and mean LCQ score was 14.6 (SD 3.0). During the study period, these PROMs did not demonstrate significant changes. Mean difference in K-BILD score between baseline and 24 weeks after start of medication was 2.3 points (95% CI − 17.5–13.0), with a higher score after 24 weeks. Mean LCQ score decreased with 0.9 points (95% CI − 4.7–6.4).

## Discussion

The current study was the first to evaluate the safety and efficacy of pirfenidone in patients with asbestosis. No novel or unexpected adverse events were noted. Home spirometry demonstrated a decline in median FVC slope before start of pirfenidone and no decline during the treatment phase. This indicates that pirfenidone might attenuate FVC decline in progressive asbestosis.

During the study, 6 patients (66.7%) reported any—mostly mild—adverse events, which is in line with real-world data in patients with IPF [13]. In a recent phase 2 trial of pirfenidone in unclassifiable ILD, the most common adverse events related to the treatment were gastrointestinal (47% in the pirfenidone group compared to 26% in the placebo group), fatigue (13% versus 10%) and rash (10% versus 7%). A large post authorization study on long-term safety of pirfenidone in IPF patients describing adverse drug reactions of special interest, reported fatigue and photosensitivity reactions/skin rashes in 24.2% and 29.0% of patients respectively [13]. In the current study, fatigue (55.6%) and rash (44.4%) were more frequently reported. A reason for this increase might be the way and frequency of AE reporting. Adverse events were actively collected using a weekly electronic questionnaire. In the current study, six patients were (temporarily) treated with a reduced dose of pirfenidone. The patients included in the current study were older (mean age of 74.7) compared to the post authorization study (mean age 69.6) and the mean baseline K-BILD score was lower than other clinical studies in IPF and progressive pulmonary fibrosis, which may reflect increased vulnerability of the current patient group [13–15]. Older age is a known risk factor associated with discontinuation of pirfenidone due to adverse drug reactions [13]. Importantly, six patients needed a dose reduction due to side effects, but all were able to continue treatment after (temporary) dose adjustment. This demonstrates the importance of patient guidance and pirfenidone dose adjustments as effective strategy to reduce side effects, so patients can continue their treatment.

Two patients died during the trial, not related to the treatment. One patient died of a respiratory cause before starting pirfenidone. The second patient discontinued pirfenidone because of worsening of his clinical situation with increasing shortness of breath despite optimal palliative care. He later died due to euthanasia. Both cases demonstrate the high vulnerability of patients with progressive asbestosis, highlighting the need for new and effective treatment.

Because asbestosis is a very rare occupational disease and only a subgroup of patients demonstrate a progressive decline in pulmonary function, inclusion of a large patient group was considered not feasible, and the study was not powered to detect any treatment effect with pulmonary function measurements. As expected, no significant changes in hospital pulmonary function (FVC, DLCO), 6 min walking test or PROMs before and after start of pirfenidone could be demonstrated in the current trial. However, we also used daily home spirometry measurements to obtain a more granular overview of FVC change over time. Recent studies showed that daily home monitoring of FVC in pulmonary fibrosis provided a sensitive prediction of disease behavior and correlated well with hospital-based measurements of pulmonary function [14, 16]. In the current study, we compared slopes of home-based FVC before and after start of pirfenidone. A significant FVC decline was found before start of pirfenidone, while FVC did not decline during the 6 month treatment phase with pirfenidone. Although the FVC slopes before and after treatment did not significantly differ in this small group size (p = 0.14), the home monitoring FVC data suggest pirfenidone reduces FVC decline in the current study with asbestosis patients with a progressive phenotype. These changes could not be captured by in-hospital measurements due to the limited sample size, which illustrates the potential value of frequent home-based measurements as exploratory endpoint in clinical trials, especially in rare diseases. Our findings are in contrast with the U-ILD study where home-spirometry failed as an endpoint due to technical and analytical reasons [12]. Nevertheless, our current study as well as previous studies show that with good instructions and technical support reliable home-spirometry can be feasible. Our findings are in line with a previous study which showed that the use of home spirometry could reduce sample sizes for future trials [17]. Besides, home monitoring can be used as a safety endpoint, as we did in the current study, with patients reporting symptoms and side-effects in an online home monitoring program. Home monitoring results were sent to the hospital in real-time, which allowed us to safely monitor at a distance, with a low burden for patients.

The main limitation of this study is the planned low number of included patients. Therefore, the primary endpoint of the study could only be descriptive, assessing safety and adverse events of pirfenidone in patients with asbestosis. Nevertheless, we believe this study adds valuable information to the field as it is the first to report prospectively on antifibrotic treatment specifically in this patient group. Moreover, it highlights the potential of online home monitoring as efficacy and safety endpoint in trials with a limited number of participants.

## Conclusions

Our study demonstrates an acceptable safety and tolerability of pirfenidone in patients with asbestosis. Additionally, this study supports the concept that antifibrotic treatment with pirfenidone may slow down disease progression in patients with asbestosis and progressive lung function decline.

## Supplementary Information

**Additional file 1.** Inclusion and exclusion criteria.

## Data Availability

The dataset used and analyzed during the current study are available from the corresponding author on reasonable request.

## Abbreviations

CI: Confidence interval
DLCO: Diffusion capacity of the lung for carbon monoxide
FVC: Forced vital capacity
6MWD: Six minute walking test
ILD: Interstitial lung disease
IPF: Idiopathic pulmonary fibrosis
K-BUILD: King’s Brief Interstitial Lung Disease
LCQ: Leicester Cough Questionnaire
NVALT: The Dutch Association of Pulmonologists
PROMs: Patient reported outcome measures

## References

[1] American Thoracic S (2004). Diagnosis and initial management of nonmalignant diseases related to asbestos. Am J Respir Crit Care Med.

[2] Yang M, Wang D, Gan S (2020). Increasing incidence of asbestosis worldwide, 1990–2017: results from the global burden of disease study 2017. Thorax.

[3] Barnikel M, Million PM, Knoop H, Behr J (2019). The natural course of lung function decline in asbestos exposed subjects with pleural plaques and asbestosis. Respir Med.

[4] Keskitalo E, Salonen J, Vahanikkila H, Kaarteenaho R (2021). Survival of patients with asbestosis can be assessed by risk-predicting models. Occup Environ Med.

[5] Noble PW, Albera C, Bradford WZ (2016). Pirfenidone for idiopathic pulmonary fibrosis: analysis of pooled data from three multinational phase 3 trials. Eur Respir J.

[6] Ley B, Swigris J, Day BM (2017). Pirfenidone reduces respiratory-related hospitalizations in idiopathic pulmonary fibrosis. Am J Respir Crit Care Med.

[7] Fan Y, Zheng C, Wu N (2020). Telomerase gene variants and telomere shortening in patients with silicosis or asbestosis. Occup Environ Med.

[8] Platenburg M, Wiertz IA, van der Vis JJ (2020). The MUC5B promoter risk allele for idiopathic pulmonary fibrosis predisposes to asbestosis. Eur Respir J.

[9] Wijsenbeek M, Cottin V (2020). Spectrum of fibrotic lung diseases. N Engl J Med.

[10] Flaherty KR, Wells AU, Cottin V (2019). Nintedanib in progressive fibrosing interstitial lung diseases. N Engl J Med.

[11] Behr J, Prasse A, Kreuter M (2021). Pirfenidone in patients with progressive fibrotic interstitial lung diseases other than idiopathic pulmonary fibrosis (RELIEF): a double-blind, randomised, placebo-controlled, phase 2b trial. Lancet Respir Med.

[12] Maher TM, Corte TJ, Fischer A (2020). Pirfenidone in patients with unclassifiable progressive fibrosing interstitial lung disease: a double-blind, randomised, placebo-controlled, phase 2 trial. Lancet Respir Med.

[13] Cottin V, Koschel D, Gunther A (2018). Long-term safety of pirfenidone: results of the prospective, observational PASSPORT study. ERJ Open Res.

[14] Moor CC, Mostard RLM, Grutters JC (2020). Home monitoring in patients with idiopathic pulmonary fibrosis. A randomized controlled trial. Am J Respir Crit Care Med.

[15] Wells AU, Flaherty KR, Brown KK (2020). Nintedanib in patients with progressive fibrosing interstitial lung diseases-subgroup analyses by interstitial lung disease diagnosis in the INBUILD trial: a randomised, double-blind, placebo-controlled, parallel-group trial. Lancet Respir Med.

[16] Moor CC, van Leuven SI, Wijsenbeek MS, Vonk MC (2021). Feasibility of online home spirometry in systemic sclerosis-associated interstitial lung disease: a pilot study. Rheumatology.

[17] Johannson KA, Vittinghoff E, Morisset J (2017). Home monitoring improves endpoint efficiency in idiopathic pulmonary fibrosis. Eur Respir J.

