# Supplementary material for: Safety and tolerability of pirfenidone in asbestosis: a prospective multicenter study
Source: Respir Res. 2022 May 28;23:139. doi: 10.1186/s12931-022-02061-2 (PMC9148498; doi:10.1186/s12931-022-02061-2)
Supplement: Supplementary file 1 — Additional file 1. Inclusion and exclusion criteria. [file 12931_2022_2061_MOESM1_ESM.docx]

## Additional file 1:

## Inclusion criteria

Patients (40-85 years) with confirmed asbestosis by Dutch NVALT IPF expertise-panel AND a history of asbestos exposition with 15-30 years latency AND

pleural plaques OR asbestos fibers in pulmonary lavage OR asbestos fibers confirmed in lung biopsy)

AND

1. written informed consent
2. FVC ≥ 50% predicted, DLCO ≥ 25%
3. Minimal 6 minute walk test distance 150 meter
4. FEV1/FVC > 0.70
5. Documented disease progression in 3-6 months (FVC decrease > 5% in < 6 months or DLCOc decrease > 10% in 3-6 months, or decrease ≥ 25 meter on 6 minute walk test in < 3-6 months)
6. > 10% interstitial fibrosis on HRCT by visual scoring

## Exclusion criteria

1. Current smoker
2. > 15% emphysema on HRCT thorax
3. Use of immunosuppressant prednisone> 10mg or other: methotrexate, azathioprine, cyclophosphamide
4. Malignancy
5. Renal impairment (GFR < 30 ml/min or dialysis)
6. Pregnancy
7. Concomitant use (<28 days) of a strong and selective inhibitor of CYP1A2 (Fluvoxamin, enoxacin)
8. History of hepatic impairment, elevation of transaminase enzymes, or the confirmation of any of the following liver function test criteria above the specified limits: Total bilirubin above the upper limit of normal (ULN), Aspartate aminotransferase (AST) or alanine aminotransferase (ALT) >1.5 × ULN, Alkaline phosphatase > 2.0 × ULN
